# NK Cells Accumulate in Infected Tissues and Contribute to Pathogenicity of Ebola Virus in Mice

**DOI:** 10.1128/JVI.01703-18

**Published:** 2019-05-01

**Authors:** H. Fausther-Bovendo, X. Qiu, S. He, A. Bello, J. Audet, G. Ippolito, G. Wong, G. Kobinger

**Affiliations:** a National Microbiology Laboratory, Public Health Agency of Canada, Winnipeg, Canada; b University of Manitoba, Winnipeg, Canada; c National Institute for Infectious Diseases, Lazzaro Spallanzani, National Institute for Infectious Diseases–IRCCS, Rome, Italy; d Department of Pathology and Laboratory Medicine, University of Pennsylvania School of Medicine, Philadelphia, Pennsylvania, USA; Icahn School of Medicine at Mount Sinai

**Keywords:** Ebola virus, lymphopenia, NK cells, ULBP-1, autologous killing

## Abstract

Understanding the immune parameters responsible for survival following Ebola virus (EBOV) infection is paramount for developing countermeasures. In lethal EBOV infections, levels of both NK and T cells decline drastically in the circulation and lymphoid tissues before death. However, the fate of these lymphocytes in viral replication sites remains unknown. In this study, reverse transcription-PCR (RT-PCR) and fluorescence-activated cell sorting (FACS) analysis were used to investigate lymphocyte frequencies in various infected mouse tissues after challenge with mouse-adapted EBOV (MA-EBOV). A decrease in NK cell numbers from systemic circulation was observed concomitant to an increase of these cells in tissues that are supporting active replication of EBOV. Unexpectedly, NK accumulation in virus replication sites correlated with enhanced EBOV disease progression in specific conditions; at a high challenge dose, NK-depleted mice displayed lower viremia and liver damage and higher hepatic T cell levels. Upregulation of UL16 binding protein 1 (ULBP-1) was detected in hepatic T cells, suggesting that NK cells participate in their elimination. Overall, this study supports the concept that NK cells accumulate in EBOV-infected tissues and can contribute to viral pathogenicity.

**IMPORTANCE** Ebola virus (EBOV) outbreaks can claim numerous lives and also devastate the local health infrastructure, as well as the economy, of affected countries. Lethal EBOV infection has been documented to decrease the levels of several immune cells in the blood that are necessary to defend the host. This decrease in immune cells is, however, not observed in individuals who survive EBOV infection. Having a better grasp of how these immune cells are lost is therefore of high importance to develop and improve new and existing therapeutics. The significance of our research is in identifying the mechanism responsible for the apparent loss of immune cells in lethal EBOV infection. This will allow therapeutic options aimed at preventing the loss of these immune cells, therefore allowing infected individuals to better fight the infection.

## Abstract (precis)

Ebola virus (EBOV) outbreaks can claim numerous lives and also devastate the local health infrastructure, as well as the economy, of affected countries. Lethal EBOV infection has been documented to decrease the levels of several immune cells in the blood that are necessary to defend the host. This decrease in immune cells is, however, not observed in individuals who survive EBOV infection. Having a better grasp of how these immune cells are lost is therefore of high importance to develop and improve new and existing therapeutics. The significance of our research is in identifying the mechanism responsible for the apparent loss of immune cells in lethal EBOV infection. This will allow therapeutic options aimed at preventing the loss of these immune cells, therefore allowing infected individuals to better fight the infection.

## INTRODUCTION

Untreated Ebola virus (EBOV) infection can lead to a hemorrhagic fever and multiple organ failure followed by septic shock, leading to death in up to 90% of cases (1). During the 2014 to 2016 EBOV outbreak, the safety and efficacy of several therapeutic and prophylactic countermeasures were clinically evaluated (2). Although the protective efficacy of the vesicular stomatitis virus (VSV) vaccine was clinically demonstrated (3), statistically significant survival benefits are yet to be demonstrated by any therapeutic efficacy trials (4, 5). Understanding the immune properties specific to surviving or succumbing to an EBOV infection is important for the optimization of therapeutic products.

Lethal EBOV infections are characterized by an increase in granulocytes and a concomitant decrease in total lymphocyte counts in the systemic circulation. Among the lymphocyte populations, the relative frequencies of both T and NK cells drastically decline in the blood of infected individuals before death. A decrease in T cell levels was documented in human infections (6), while depletion of both T and NK cells was observed in nonhuman primates (NHPs) (7, 8) and rodents (9) infected with EBOV. Interestingly, this decrease was only observed in cases of lethal human infection but not in human survivors (6). This is of particular interest, as both NK and T cells are important players in EBOV clearance (10–12).

The mechanism responsible for the decline in circulating T and NK cells is suspected to be indirect, since neither T nor NK cells are infected by EBOV (7, 13). Apoptosis is currently believed to be one of the possible mechanisms responsible for the loss of T and NK cells. Numerous mechanisms, such as FAS/CD95- (6, 7, 14), TNF-related apoptosis-inducing ligand (TRAIL)- (7), and superantigen (15)-mediated cell death, have been proposed to explain T and NK cell apoptosis.

During EBOV infection, lymphocyte apoptosis has mainly been studied in peripheral blood and lymphoid tissues, including the spleen and lymph nodes of infected animals. (6–9, 16). However, the lymphopenia observed in peripheral blood and lymphoid tissues might not reflect the fate of lymphocyte populations in nonlymphoid tissues, such as the liver, lung, and kidney, where EBOV also replicates. T and NK cells migrate to infection sites in response to chemokine secretion. In addition, tissue-resident T and NK cell populations have been described in various nonlymphoid tissues, including the skin, liver, gut, uterus, and lungs (17, 18). Tissue-resident lymphocytes and their circulating counterparts often have distinct phenotypic and functional properties (19, 20), which could result in these pools of lymphocytes having different fates during an EBOV infection.

This study documents the fate of lymphocyte populations in various viral replication sites. This study also investigates the contribution of NK cell-mediated killing of immune cells to EBOV-induced lymphopenia. Here, both flow cytometry (fluorescence-activated cell sorting [FACS]) and reverse transcription-PCR (RT-PCR) were used to monitor the presence of NK cells in the blood and tissues of mice infected with mouse-adapted EBOV (MA-EBOV) when lymphopenia was detectable. Depletion studies of NK cells in mice were performed to investigate NK cell function during MA-EBOV infection. The level of activating NK ligands was also monitored in MA-EBOV-infected tissues to inform about putative bystander NK cell targets.

## RESULTS

### NK cells accumulate in viral replication sites during MA-EBOV infection.

**FIG 1:**
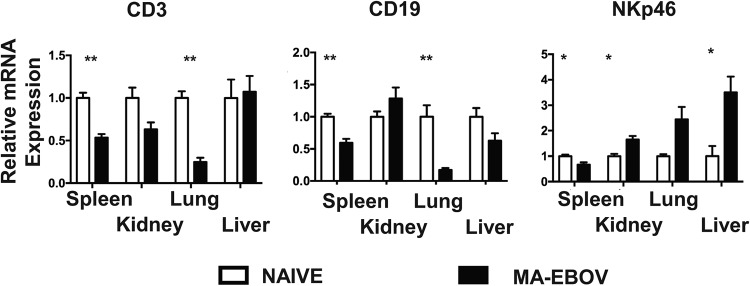
NK cell accumulation in MA-EBOV-infected tissues. Levels of CD3Ɛ, CD19, and NKp46 mRNA were compared using RT-PCR of spleen, kidney, lungs, and liver from naive and MA-EBOV-infected (100 × LD_50_) mice 4 days after infection. Six mice were used per group, and actin was used to normalize mRNA for each sample. Relative mRNA expression levels (mean ± standard error of the mean [SEM]) are indicated.

**FIG 2:**
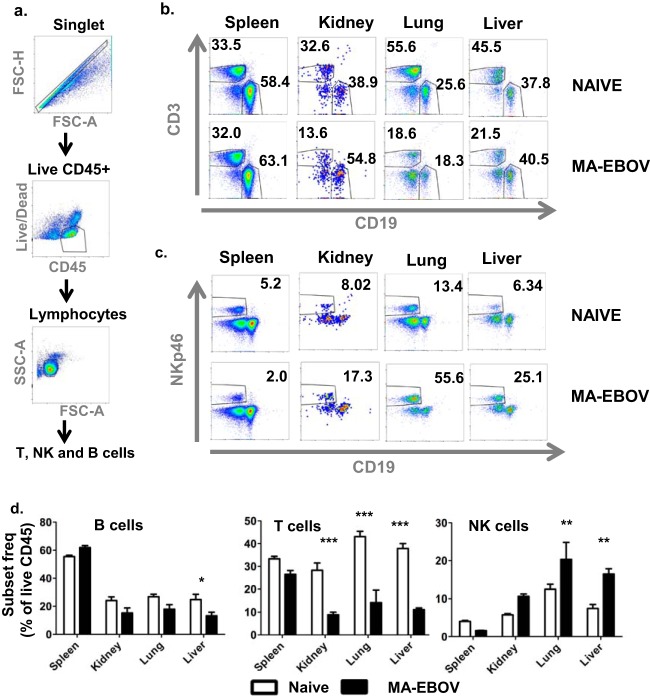
NK cell frequencies increased in MA-EBOV-infected tissues. Frequencies of live (live/dead low) B (CD45^+^ CD19^+^), T (CD45^+^ CD3^+^), and NK (CD45^+^ NKp46+) cells in spleen, kidney, lung, and liver were measured by FACS in naive and MA-EBOV-infected (100 × LD_50_) mice 4 days after infection. (a) Gating strategies for B, T, and NK cell frequencies. Representative plots of T, B (b), and NK cell (c) frequencies from the lymphocyte gate are illustrated. (d) Summary graphs (*n* = 6 per group) of B, T, and NK cell frequencies (mean ± SEM) are shown.

We sought to examine the fate of lymphocytes outside of the peripheral blood and lymphoid tissues during MA-EBOV infection. First, the kinetics of lymphopenia in peripheral blood were monitored in mice infected with a lethal dose of MA-EBOV (100 × 50% lethal dose [LD_50_]). As previously reported, the total lymphocyte percentage started declining 3 days postinfection and remained low, around 30%. Similarly, total lymphocyte counts decreased from 3 days postchallenge but started rising 5 days postinfection (data not shown). Based on the above kinetics, the presence of T and NK cells was then monitored in subsequent experiments by RT-PCR and FACS in various tissues 4 days post MA-EBOV (100 × LD_50_) challenge. The entire B cell population was used as a control, as its frequency within the lymphocyte population remains relatively stable during EBOV infection. Mice were either mock infected or infected with MA-EBOV. Four days postinfection, the spleen, kidney, lung, and liver were collected. The frequencies of T, NK, and B cells were first analyzed by RT-PCR. mRNA levels were normalized with actin to ensure that the loss of a specific immune subset would not artificially increase the frequency of other subsets. CD3Ɛ and CD19 mRNA levels decreased in all tissues assessed except for the liver and kidney, where no statistically significant changes in CD3Ɛ and CD19 mRNA levels were observed (Fig. 1), thus confirming the overall lymphopenia. In contrast, the NKp46 mRNA level decreased in the spleen of MA-EBOV-infected mice, while its level rose 1.7, 2.4, and 3.5-fold, respectively, in the kidney, lung, and liver of infected mice (Fig. 1), indicating an accumulation of NK cells to these organs at that time point. To further document the frequency of T, NK, and B cells *in vivo*, each respective population was measured by FACS in the above-mentioned tissues of MA-EBOV- and mock-infected mice. Within all tested tissues, there was no significant difference in the frequency of live hematopoietic cells (live CD45^+^), which include both lymphocytes and granulocytes. As a result, frequencies of other immune subsets were expressed as the percentage of live CD45^+^ cells. The frequencies of T and NK cells decreased, while the frequency of B cells slightly increased, in the spleen of MA-EBOV-infected mice. In the remaining MA-EBOV-infected tissues (liver, lungs, and kidney), there was a trend toward a lower B cell frequency and a sharp decline in T cell frequency. Conversely, in all these sites, the frequency of NK cells significantly increased, suggesting an NK cell accumulation within these tissues (Fig. 2). Taken together, both FACS and RT-PCR results suggest that NK, but not T, cells accumulate in MA-EBOV-infected tissues, also indirectly reinforcing previous studies indicating that T cell depletion is due to cell death.

### Levels of IFN-α and KC, which modulate NK cell migration, are elevated after MA-EBOV infection.

**FIG 3:**
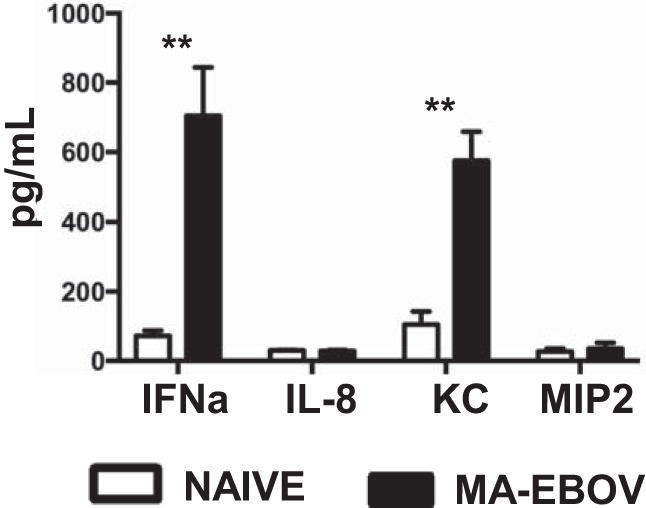
IFN-α and KC are induced after MA-EBOV challenge. Circulating level (mean ± SEM) of various cytokines were measured by ELISA in sera of naive (open bars) and MA-EBOV-infected (100 × LD_50_) mice (black bars) 4 days postchallenge. Results are representative of a single experiment with 8 mice per group.

During hepatitis B virus (HBV) and murine cytomegalovirus (MCMV) infection, NK accumulation in the liver is dependent on interferon alpha (IFN-α) and IL-8 production (21, 22). To investigate a possible role of these cytokines in promoting NK cell accumulation in the liver during MA-EBOV replication, circulating levels of IFN-α, IL-8 and its murine homologue KC/CXCL1 (KC), and MIP2/CXCL2 (MIP2) (23) were measured by enzyme-linked immunosorbent assay (ELISA) 4 days postchallenge in mice. Elevated levels of IFN-α and KC, but not IL-8 or MIP2, were observed in sera from MA-EBOV-challenged mice (Fig. 3). These results suggest that elevated IFN-α and KC production could both contribute to NK cell accumulation in the liver during MA-EBOV infection.

### The role of NK cells during EBOV infection is dependent on viral load.

**FIG 4:**
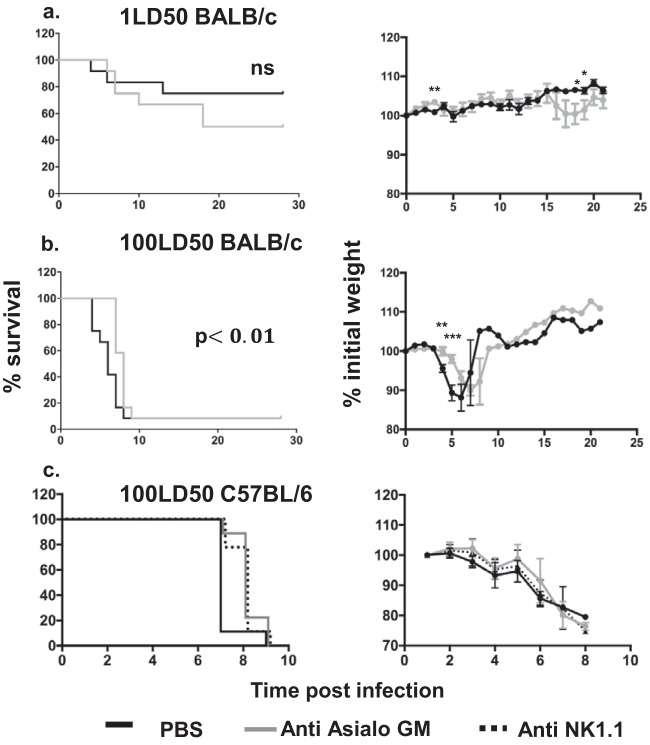
NK cells can have beneficial or detrimental roles depending on MA-EBOV infectious dose. BALB/c (a and b) and C57BL/6 mice (c) were treated with PBS (black lines) or one of two NK-depleting antibodies, anti-asialo GM1 (gray lines) or anti-NK1.1 (PK136) (dotted lines). Survival curves (left) and weight loss (right) are illustrated. (a and b) BALB/c mice (*n* = 12 per group) were infected with a low dose (1LD50) (a) or high dose (100LD50) of MA-EBOV (b) and monitored for 28 days postinfection. (c) C57BL/6 mice were infected with MA-EBOV (100LD50) and monitored postinfection. ns, non-statistically significant difference (*P* > 0.05).

To better understand the role of NK cells during EBOV infection, NK depletion studies in BALB/c mice were performed using anti-asialo GM1 antibodies. NK-depleted mice were infected with either a low dose (1 × LD_50_) or a high dose (100 × LD_50_) of MA-EBOV. At low viral load, fewer NK-depleted mice survived the challenge compared with their mock-depleted counterparts (Fig. 4a). This suggests that NK cells can interfere with the progression of MA-EBOV-induced disease in these conditions. Interestingly, in mice infected with a 100-fold higher dose of MA-EBOV, no difference in survival rate was observed with or without NK cell depletion. However, NK-depleted mice had delayed times to symptom (weight loss) onset and death than the control mice, 7.6± 0.2 days versus 5.8 ±0.4 days, (*P* < 0.001) (Fig. 4b). Anti-asialo GM1 antibodies have been reported to deplete both NK and basophils (24). To ensure that the detrimental effect observed was due to NK cells, the latter challenge experiment was repeated in C57BL/6 mice using two distinct NK-depleting antibodies. Both anti-asialo GM1 and anti-NK1.1 delay the mean time to death of MA-EBOV-infected (100 × LD_50_) mice compare with mock-treated ones from 7.2 to 8.1 and 7.9 days postchallenge, respectively (Fig. 4c). This delayed time to death suggests that with higher initial viral load, the NK cell response can be detrimental to the host. Interestingly, in the mouse model of lymphocytic choriomeningitis virus (LCMV) infection, NK cells differentially affect the host immune response, depending on the challenge dose (25).

### NK depletion delays liver damage during MA-EBOV infection.

**FIG 5:**
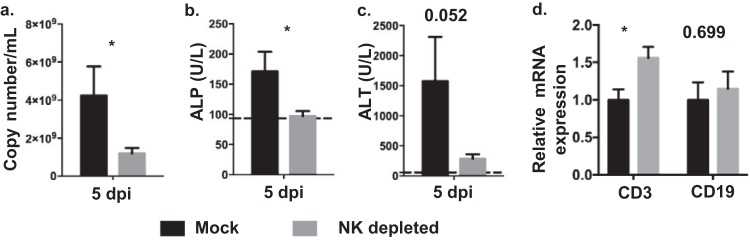
NK cells contribute to MA-EBOV pathogenicity. (a to c) Mock- (black) and NK-depleted mice (gray) were infected with a high dose (100 × LD_50_) of MA-EBOV. Five days postchallenge, viremia (a), ALP (b), and ALT (c) were measured by RT-PCR and using a VetScan VS2 instrument, respectively (*n* = 8 per group). Means ± SEM are shown; dotted lines represent the average values from naive mice (*n* = 6). (d) Levels of CD3Ɛ and CD19 mRNA were compared by RT-PCR between mock- and NK-depleted mice challenged with MA-EBOV (*n* = 6 per group). Relative mRNA levels (mean ± SEM) are illustrated. *P* values are indicated where the differences fell short of statistical significance. NK depletion was achieved by injecting anti-asialo GM1 antibodies.

To investigate the mechanism behind NK cell-mediated disease aggravation, viral load and liver damage were monitored in mock- and NK-depleted mice infected with MA-EBOV (100 × LD_50_). Based on elevated alanine aminotransferase (ALT) and alkaline phosphatase (ALP) levels, no significant liver damage was detectable 4 days post MA-EBOV challenge. As a result, the above parameters were assessed 5 days postchallenge. Viremia, ALP, and ALT levels were all greatly reduced (*P* values of 0.04, 0.02, and 0.05 respectively) in NK-depleted mice (Fig. 5a to c), further supporting the idea that NK cells can play a detrimental role in specific conditions related to Ebola virus replication.

Both T and B cells are involved in controlling viremia during EBOV infection (12, 26, 27). To probe the decreased viremia and liver damage in NK-depleted mice, hepatic levels of both T and B cells were compared by RT-PCR between mock- and NK-depleted mice infected with MA-EBOV. Although no difference in hepatic B cell level was detectable, there was on average a 1.56-fold increase in the hepatic T cell level in NK-depleted mice compared with that in their mock-depleted MA-EBOV-infected counterpart (Fig. 5d). This result may indicate a direct or indirect pathogenic effect of NK cells toward hepatic T cells.

### ULBP-1 is overexpressed by hematopoietic cells in the liver of MA-EBOV-infected mice.

**FIG 6:**
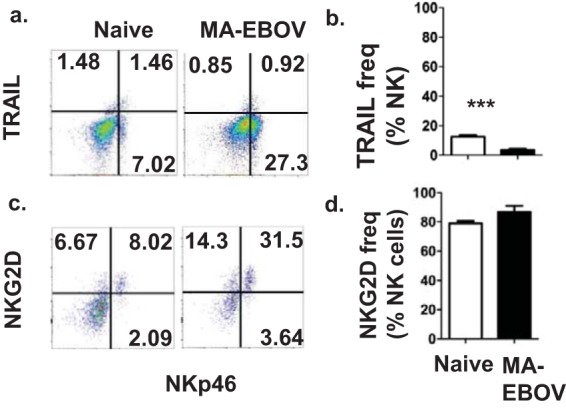
Abundant NKG2D but limited TRAIL expression was detected on liver NK cells from MA-EBOV-infected mice. On day 4 after MA-EBOV infection (100 × LD_50_), TRAIL (a and b) and NKG2D (c and d) surface expression on NK cells were measured by FACS, with naive mice used as negative controls. Representative (a and c) and summary graphs (*n* = 6/group; mean ±SEM) (b and d) are illustrated.

**FIG 7:**
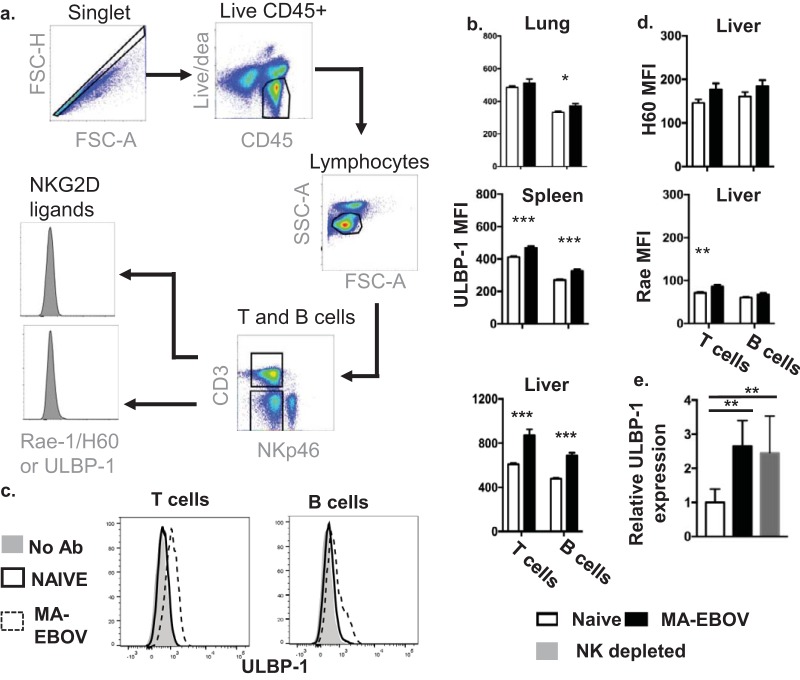
ULBP-1/MULT-1 is upregulated in the liver on MA-EBOV-infected mice. Levels of NKG2D ligands (ULBP-1, H60, and Rae-1) were measured by FACS on live T (live CD45^+^ CD3^+^ NKp46^−^) and B cells (live CD45^+^ CD3^−^ NKp46^−^) from spleen, lung, and liver of naive or MA-EBOV-infected mice (100 × LD_50_) 4 days postchallenge. (*n* = 6/group). (a) Gating strategies for H60, Rae-1, and ULBP-1 expression levels. (b) ULBP-1 expression (mean ± SEM) on T and B cells from the spleen, lungs, and liver of mock- and MA-EBOV-infected mice are depicted. Summary graphs (b), as well as representative plots (c), are displayed for ULBP-1 (*n* = 9/group). (d) H60 and Rae-1 levels (mean ± SEM) on hepatic lymphocytes are illustrated. (e) Changes in ULBP-1 mRNA were also measured by RT-PCR between naive and MA-EBOV-infected mice (mock- or NK-depleted). Relative mRNA levels (mean ± SEM) are illustrated. NK depletion was achieved by injecting anti-asialo GM1 antibodies.

The phenomenon of NK cell-mediated pathogenicity was further investigated. We hypothesized that NK cell killing of hepatic T cells in MA-EBOV-infected mice was responsible for their detrimental effects at higher loads of MA-EBOV. Unfortunately, increased NK cell killing of hepatic T cells from MA-EBOV-infected mice could not be directly demonstrated using *in vitro* killing assays due to the limited number of lymphocytes which could be isolated from livers. Instead, expression of activating NK receptors and ligands was monitored on hepatic NK and T cells, respectively. Surface expression of activating TRAIL receptors or activating NKG2D ligands is sufficient for target cells to become sensitive to lysis by autologous NK cells expressing TRAIL or NKG2D, respectively. Levels of NKG2D and TRAIL were first monitored in hepatic NK cell by FACS in mock- or MA-EBOV-infected mice (100 × LD_50_). In MA-EBOV-infected mice, only 5% of NK cells in the liver expressed TRAIL on their surface (Fig. 6). In contrast, an average of 90% of NK cells expressed NKG2D in the liver of MA-EBOV-challenged mice (Fig. 6). Due to high level of NKG2D expression on NK cells, the amount of NKG2D ligands on T and B lymphocytes from the liver was measured in mock- or MA-EBOV-infected mice (100 × LD_50_). Of note, activating TRAIL receptors level were not measured on immune cells due to the low expression levels of TRAIL on NK cells. There was little to no increase in the surface expression of H60 and Rae-1, two NKG2D-activating ligands, on T and B cells in the liver of infected mice compared to that in their naive counterparts. In contrast, surface expression of UL16 binding protein (ULBP-1), a third NKG2D-activating ligand, was significantly increased on the surface of T and B cells in the liver but not in other tested tissues from infected mice (Fig. 7a and d). To confirm the above FACS results, ULBP-1 mRNA levels were compared between naive and MA-EBOV-infected mice (100 × LD_50_). Similar to the FACS results, there was a wide range of ULBP-1 induction levels in infected mice; overall, there was a 2.66-fold increase in ULBP-1 expression in the liver of MA-EBOV-infected mice compared with that in naive mice (Fig. 7e). It is worth pointing out that there was no difference in the ULBP-1 mRNA level between mock- and NK-depleted mice post MA-EBOV-challenge (Fig. 7e). ULBP-1 is probably induced by direct contact with MA-EBOV viral particles or by MA-EBOV-induced inflammation. Despite reduced NK killing of ULBP-1 cells, lower ULBP-1 induction in NK-depleted mice is probably responsible for the similar ULBP-1 expression levels of mock- and NK-depleted mice after MA-EBOV challenge.

ULBP-1 induction in T cells has previously been shown to render them sensitive to autologous NK cell-mediated killing (28). Therefore, the above results suggest that NK killing of ULBP-1-positive T cells in the liver may contribute to MA-EBOV immune evasion and viral pathogenicity.

## DISCUSSION

NK cells possess multiple antiviral properties. They can kill infected cells, secrete inflammatory cytokines, and shape the adaptive response by interacting with dendritic cells (29–31). Accordingly, the beneficial impact of NK cells during MA-EBOV infection has previously been demonstrated in mice treated with virus-like particles (VLPs) or recombinant vesicular stomatitis virus encoding EBOV glycoprotein (VSV-GP) 1 to 3 days before or after challenge, respectively (11, 13).

The present study indicates that NK cell depletion in peripheral blood and the spleen of MA-EBOV-infected mice was concomitant with an accumulation of NK cells in nonlymphoid tissues. The sharpest increase in NK cell frequency was observed in the liver, which is a major replication site early after EBOV infection (7). This observation suggests that NK cell accumulation may directly correlate with viral load and EBOV-mediated inflammation. NK cells migrate in an IFN-α- and IL-8-dependent manner to the liver of HBV- and MCMV-infected individuals and mice, respectively (21, 22). MA-EBOV infection induces secretion of IFN-α and KC, the murine IL-8 homolog, suggesting that NK accumulation might results from circulating NK migration to replication sites. It is worth pointing out that proliferation of tissue-resident NK cells may also contribute to NK accumulation. Due to the lack of specific markers to differentiate tissue resident from circulating NK cells in our study, the contribution of each mechanism cannot be determined. Interestingly, a decrease in the number of circulating NK cells has also been described in NHPs infected with Lassa virus (32), suggesting that vast NK cell accumulation in sites of viral replication might be a common feature of viral hemorrhagic fevers.

Although the antiviral properties of NK cells are well documented, data obtained in this study indicate that the activity of NK cells can also be detrimental to the host. Infection of perforin-deficient mice previously suggested that killing of noninfected (bystander) cells may be involved in MA-EBOV disease aggravation (13). Here, NK depletion studies, in conjunction with the significant increase in ULBP-1 surface expression on T cells in the liver of MA-EBOV-infected mice, suggest NK cell killing of T cells. Although sufficient T and NK cells could not be obtained from liver to demonstrate *in vitro* NK killing of hepatic T cells from MA-EBOV-infected mice, Cerboni and colleagues (28) previously demonstrated NK killing of ULBP-1-positive T cells. Indeed, *in vitro* T cell activation leads to a similar increase in ULBP-1 surface expression, as well as an increase in susceptibility to autologous NK cells lysis via NKG2D (28). It is worth noting that NK killing of T lymphocytes within the liver of infected mice may contribute to MA-EBOV immune evasion. T cells are recruited to infection sites independently of antigen specificity (33, 34). Even though the role of these non-antigen-specific infiltrating cells is not fully understood, they are thought to participate in controlling invading pathogens by producing antiviral cytokines in response to the inflammatory cytokine milieu (35). Therefore, NK killing of T cells within the liver of infected mice, independently of their antigen specificity, may facilitate viral replication and therefore exacerbate liver damage, as MA-EBOV replication in hepatocytes leads to necrosis.

Additional mechanisms may also be involved in the observed NK cell-mediated MA-EBOV disease exacerbation. Indeed, no significant ULBP-1 induction on T cells was detected in the lungs of MA-EBOV-infected mice despite a reduction in pulmonary T cell frequency. In addition, MA-EBOV replicates heavily in the liver but not in the lungs of intraperitoneally challenged mice, resulting in substantially more immune activation, including cytokines in the liver. Taken together, the above observations suggest that distinct mechanisms in the lung versus the liver of MA-EBOV-infected mice could be at play.

NK killing of infected hepatocytes and epithelial cells could aggravate liver damage, while NK killing of infected antigen-presenting cells, such as dendritic cells and macrophages, might dampen the cellular response against MA-EBOV. In addition, T cell apoptosis due to improper activation rather than direct killing by NK cells may also be responsible for the decrease in T cell frequency in MA-EBOV-infected mice. Of note, 5 days post MA-EBOV infection, levels of virus-specific IgG are very low. As a result, NK-mediated antibody-dependent cellular cytotoxicity (ADCC) is not expected to contribute significantly to the disease aggravation observed in our experiments.

Together, our work suggests the following model, in which, after low MA-EBOV challenge dose, NK cells participate in viral clearance and therefore improve survival. In contrast, in untreated mice challenged with a high dose of MA-EBOV, NK cells contribute to viral pathogenicity. Treatments such as VLP or VSV-GP administration close to the time of challenge also promote a beneficial role of NK cells in mouse challenge with a high infectious dose by reducing the initial viral load (11, 13).

Data presented in this manuscript are restricted to the mouse model of EBOV infection and would therefore need confirmation in EBOV-infected individuals. However, data from EBOV-infected NHPs and individuals indicates that NK cell-mediated disease aggravation is probably not limited to the rodent model of EBOV infection. An increase in circulating IFN-α levels in NHPs challenged with a lethal dose of EBOV was previously reported (7). Wauquier and colleagues (36) also demonstrated that KIR2DS1 and KIR2DS3, two activating killer immunoglobulin-like receptors (KIR), which are expressed on NK cells and a subset of T cells, were more frequent in lethal EBOV infections than in EBOV survivors and the noninfected population. Whether additional NK-activating ligands, such as NKp44L and NKp30L, which do not have known murine equivalents (37), are also involved in disease aggravation in EBOV-infected individuals will require further investigation. It is worth noting that NK cell disease aggravation has been previously described in the context of other infections. In the mouse model of LCMV infection, viral challenge also induces expression of NKG2D ligands on T cells. Furthermore, NK depletion resulted in lower viremia, liver damage, and virus-specific T cell response (38). NK cells also exacerbate disease progression in Semliki Forest virus- (39) and Streptococcus pneumonia (40)-infected mice. In addition, NK cell killing of CD4 T cells has also been postulated to participate in the decline of CD4 T cells in HIV-1 infection (41, 42).

Overall, the evidence presented here supports the idea that the decrease in NK cell numbers from the systemic circulation is associated with an NK cell accumulation in nonlymphoid tissues supporting EBOV replication. This study also indicates that relocated NK cells can participate in MA-EBOV pathogenicity under specific conditions. Early reduction of initial viral load would influence the balance of NK cell-mediated functions and could led to better clinical management of infected individuals.

## MATERIALS AND METHODS

### Mice and virus strains.

Five- to 6-week-old female BALB/c and C57BL/6 mice were purchased from Charles River (Quebec, Canada). Mice were infected with either 100 × LD_50_ (high dose) or 1 × LD_50_ (low dose) of mouse-adapted (MA) EBOV. The MA-EBOV strain used has been previously described (43) and is derived from the Mayinga strain of EBOV.

### Antibodies.

CD3 PE (500A2), NKp46 BV605 (29A1.4), CD19 PercPCy55 (1D3), CD45 alexa700 and CD45 APC-Cy7 (30-F11), CD8 PE-Cy7 (53-6.7), and CD4 BV605 (RM4-5) antibodies were purchased from BD Biosciences (San Jose, CA), while CD3-PeCy7 (17A2), NKP46-FITC (29A1.4), and TRAIL-PE (N2B2) were from BioLegend (San Diego, CA). The fixable viability dye eFluor 506 (eBioscience, San Diego, CA) or Live/Dead fixable far red dead cell stain kit (Life Technologies, Burlington, Canada) were used to exclude dead cells.

### Flow cytometry.

Spleen, lungs, liver, and kidneys from infected mice were harvested 4 days post MA-EBOV infection (100 × LD_50_). Corresponding tissues from healthy mice were used as a control. Tissues were homogenized using a plunger, and a single cell suspension was obtained after filtering the homogenate through a 22-μm cell strainer. After extensive washes with phosphate-buffered saline (PBS), samples were stained with the designated antibody cocktail and viability dye to exclude dead cells.

After staining, all samples were analyzed using an LSR II flow cytometer (BD Biosciences, San Jose, CA). Analysis was performed using Flow Jo software (TreeStar, Ashland, OR).

### RT-PCR.

Spleen, lungs, liver, and kidneys from naive or infected mice were harvested 4 days post mock or MA-EBOV infection (100 × LD_50_), respectively, and preserved in RNAlater (Qiagen, Toronto, Canada) until use. RNA was purified from 30 mg of each tissue using the RNeasy kit (Qiagen) according to the manufacturer's instructions. One µg of purified RNA was transcribed into cDNA using the High-Capacity RNA to cDNA kit (Life Technologies, Burlington, Canada) according to the manufacturer's instructions. Following reverse transcription (RT), 1 µl of cDNA was analyzed by PCR using the TaqMan gene expression master mix (Life Technologies) and with the following forward (F) and reverse (R) primers and probes (P) in the 5′ to 3′ orientation; CD3Ɛ (F-GCCCAGAGGGCAAAACAAG; R-TGCGGATGGGCTCATAGTCT; P-AGCGGCCACCACCTGTTCCCA), CD19 (F-CGCCAGGAGATTCTTCAAAGTG; R-AGAGCACATTCCCGTACTGGTT; P-CCTCCCTCGGGAAACGGGACC), and NKP46 (F-GACTCTCCCGAAACCCATCA; R-GTTCACCGAGTTTCCATTTGTG; P-TGGGCCAAACCCAGCATCATGG). The beta-actin (Mm01205647_g1) primer/probes mix was from Life Technologies. PCR plates were run on a StepOnePlus thermocycler (Applied Biosystems, Burlington, Canada). CD3Ɛ, CD19, and NKp46 mRNA levels were used to monitor T, B, and NK cell frequencies, respectively, by RT-PCR, while beta-actin mRNA was used as a loading control. Fold changes between tested mice were calculated based on the threshold cycle (ΔΔ*C_T_*) method.

For ULBP-1 quantification, RT-PCR analysis was performed as described above, except ULBP-1 (Mm_ULBP1_1_SG) and actin-beta (Mn_Actb_1_SG) Quantitect primers were obtained from Qiagen, and the PCRs were performed using the iQ SYBR green supermix (Bio-Rad, Mississauga, Canada) according to the manufacturer’s instructions.

### NK depletion studies.

For NK cell depletions, mice received either 40 µl of polyclonal rabbit anti-asialo GM1 antibodies (Wako BioProducts, Richmond, VA) 1 day before MA-EBOV challenge and on days 5, 11, and 18 postinfection or 100 μg of anti-mouse NK1.1 (PK136) antibodies (Cedarlane, Burlington, Canada) 2 days before and on the day of challenge, as previously reported (44). Both NK-depleted mice and control mice were monitored daily for survival, weight loss, and signs of disease for 28 days after MA-EBOV infection. NK cell depletion efficiency was confirmed by FACS using unchallenged mice.

### Viremia.

Mice were bled via the retro-orbital route into EDTA tubes (BD Biosciences, San Jose, CA). Viremia was determined by RT-qPCR as previously reported (45). Briefly, RNA was extracted using QIAmp Viral RNA mini kit (Qiagen). Levels of EBOV RNA polymerase were detected by RT-qPCR.

### Blood counts and liver damage.

Mice were bled via the retro-orbital route and sera were collected using serum separation tubes (BD Biosciences). Blood counts were measured using a VetScan HM5 instrument (Abaxis Veterinary Diagnostics, Union City, CA), while alanine aminotransferase (ALT) and alkaline phosphatase (ALP) levels were measured using a VetScan VS2 instrument (Abaxis Veterinary Diagnostics).

### Cytokine serum levels.

IFN-α and IL-8 ELISA kits were purchased from eBioscience, Inc., and MyBioSource, while KC and MIP2 ELISA kits were from Cedarlane. Serum levels were measured according to the manufacturers’ instructions.

### Ethics statement.

All infectious animal work was performed in the biosafety level 4 biocontainment laboratory at the Public Health Agency of Canada. The procedures described in this manuscript were approved under animal use document H13-009 by the Canadian Science Centre for Human and Animal Health Care Committee, following the guidelines of the Canadian Council on Animal Care. Animals were all acclimatized for at least 7 days prior to the start of any experiments. Animals were fed and monitored daily before and during the course of the experiments.

### Statistical analysis.

Differences in immune cell frequencies were analyzed using a two-way ANOVA test followed by a Bonferroni test, while differences in mRNA levels, NKG2D ligands, weight loss, cytokine levels, ALT, ALP, and viremia were analyzed using unpaired *t* tests. For RT-PCR results, the mRNA level of each sample was first normalized using actin (using the ΔΔ*C_T_* method), as previously described (46). All statistical analyses were performed using GraphPad Prism version 5.03 software. Throughout this article, *, **, and *** indicate statistically significant differences, with *P* values of <0.05, 0.01, and 0.001 respectively, while ns indicates a non-statistically significant difference (*P* > 0.05).
